# A195 MALNUTRITION IMPAIRS WOUND HEALING DURING DSS COLITIS AND AN IN-VITRO EPITHELIAL MIGRATION ASSAY

**DOI:** 10.1093/jcag/gwae059.195

**Published:** 2025-02-10

**Authors:** P Littlejohn, H Yang, K Wang, M Kobor, B vallance

**Affiliations:** Pediatrics, The University of British Columbia, Vancouver, BC, Canada; Pediatrics, The University of British Columbia, Vancouver, BC, Canada; Pediatrics, The University of British Columbia, Vancouver, BC, Canada; Pediatrics, The University of British Columbia, Vancouver, BC, Canada; Pediatrics, The University of British Columbia, Vancouver, BC, Canada

## Abstract

**Background:**

Malnutrition is a major public health burden. Caused by insufficient intake of food/nutrients, malnutrition often stems from poverty and lack of access to food. Malnutrition is also a common complication of many gastrointestinal diseases, including inflammatory bowel disease (IBD). Reportedly, more than half of all IBD patients suffer from micronutrient or macronutrient malnutrition, resulting from malabsorption, low nutrient intake, and excessive bleeding or diarrhea. Nutritional deficiencies can alter the course of IBD, prolong hospitalizations, alter treatment response, and delay wound healing. Notably, epithelial wound healing is critical in IBD, but efficient re-epithelization rely on nutrients such as zinc and vitamin A. I thus **hypothesize** that malnutrition will impair intestinal epithelial wound repair, in vivo and in vitro.

**Aims:**

We tested how malnutrition affects wound healing following epithelial damage caused by dextran sulphate sodium (DSS)-induced colitis and in a 2D monolayer gap closure assay.

**Methods:**

We used our previously published mouse model of multiple micronutrient deficiencies (MND) and induced colitis using dextran sodium sulfate (DSS). Weanling three-week-old male C57BL/6N mice were fed a diet deficient in (zinc, vitamin A, B12, folate and iron) or a control diet for 4 weeks. At 3 weeks, we induced colitis by administering 2.5% DSS in drinking water for 4 days, plus 3 days of water recovery. We investigated epithelial damage, wound repair and molecular responses through histological and gene expression profiling. We also generated 3D colonoids from non-colitic malnourished mice, converted them to a 2D monolayer and subjected them to a wound migration assay cultured using an established malnourished media containing standard nutrients, and 50% of normal levels.

**Results:**

We found that while DSS induced tissue damage both in our control and MND mice, those on the MND diet exhibited impaired tissue repair with decreased re-epithelization over the wounded area compared to controls. Organoids cultured in the 50% malnourished media showed diminished growth, being smaller and less dense compared to standard media. Notably, epithelial migration towards gap closure in the 2D monolayer was delayed by 75% compared to standard media.

**Conclusions:**

Our work demonstrates that malnutrition impairs intestinal wound repair mechanisms. We also demonstrate an effective organoid model for examining the effects of malnutrition in vitro. We propose that efforts to address underlying malnutrition may improve wound healing leading to better outcomes for patients with IBD.

**Funding Agencies:**

CIHRTRIANGLE/Crohn’s and Colitis Canada, BC Children’s Hospital Research Foundation

